# 

**Published:** 1860-07

**Authors:** 


### CONTENTS.

### ORIGINAL COMMUNICATIONS.

PAGE.
ARTICLE I.—An Essay read before the Medical Association of the State of Georgia, at their Annual Meeting, at Rome, Ga., April 11th, 1860. By ROBERT SOUTHGATE, A. M., M. D. "*Nil remedium, nisi tempestivo usu sit.*" .......... 641
ARTICLE II.—Phrenology; By J. W. CLIFT, Columbus, Ga., ........ 683

### EDITORIAL AND MISCELLANEOUS.

American Medical Association. Thirteenth Annual Meeting, held at New Haven, June 5th, 6th and 7th, 1860, .......... 688
American Medical Association and Convention of Medical Teachers, . 701
Metropolitan and Central Medical Ethics, .......... 702
Medical Journalism, .......... 703
Meteorological Observations, .......... 704

